# Selective Serotonin Reuptake Inhibitors and Gastrointestinal Bleeding: A Case-Control Study

**DOI:** 10.1371/journal.pone.0019819

**Published:** 2011-05-18

**Authors:** Alfonso Carvajal, Sara Ortega, Lourdes Del Olmo, Xavier Vidal, Carmelo Aguirre, Borja Ruiz, Anita Conforti, Roberto Leone, Paula López-Vázquez, Adolfo Figueiras, Luisa Ibáñez

**Affiliations:** 1 Instituto de Farmacoepidemiología, Universidad de Valladolid, Valladolid, Spain; 2 Department of Pharmacology, Therapeutics and Toxicology, Universitat Autònoma de Barcelona, Fundació Institut Català de Farmacologia, WHO Collaborating Centre for Research and Training in Pharmacoepidemiology, Barcelona, Spain; 3 Clinical Pharmacology Service, Hospital Universitari Vall d'Hebron, Barcelona, Spain; 4 Pharmacovigilance Unit, Galdakao-Usansolo Hospital, Bizkaia, Spain; 5 Servizio di Farmacologia Medica, Università de Verona, Italy; 6 Departmento de Medicina Preventiva y Salud Pública, Universidad de Santiago de Compostela, Santiago de Compostela, Spain; 7 Consorcio de Investigación Biomédica en Epidemiología y Salud Pública (CIBER en Epidemiología y Salud Pública - CIBERESP), Spain; University of British Columbia, Canada

## Abstract

**Background:**

Selective serotonin reuptake inhibitors (SSRIs) have been associated with upper gastrointestinal (GI) bleeding. Given their worldwide use, even small risks account for a large number of cases. This study has been conducted with carefully collected information to further investigate the relationship between SSRIs and upper GI bleeding.

**Methods:**

We conducted a case-control study in hospitals in Spain and in Italy. Cases were patients aged ≥18 years with a primary diagnosis of acute upper GI bleeding diagnosed by endoscopy; three controls were matched by sex, age, date of admission (within 3 months) and hospital among patients who were admitted for elective surgery for non-painful disorders. Exposures to SSRIs, other antidepressants and other drugs were defined as any use of these drugs in the 7 days before the day on which upper gastrointestinal bleeding started (index day).

**Results:**

581 cases of upper GI bleeding and 1358 controls were considered eligible for the study; no differences in age or sex distribution were observed between cases and controls after matching. Overall, 4.0% of the cases and 3.3% of controls used an SSRI antidepressant in the week before the index day. No significant risk of upper GI bleeding was encountered for SSRI antidepressants (adjusted odds ratio, 1.06, 95% CI, 0.57–1.96) or for whichever other grouping of antidepressants.

**Conclusions:**

The results of this case-control study showed no significant increase in upper GI bleeding with SSRIs and provide good evidence that the magnitude of any increase in risk is not greater than 2.

## Introduction

Acute upper gastrointestinal (GI) bleeding is a prevalent and clinically significant condition with important implications for health care costs worldwide. In the United States, more than 400,000 hospital admissions per year for upper GI bleeding are estimated to occur, and mortality ranges between 3% and 14% [1]; this has not changed in the past 10 years and increases with increasing age. Known risk factors for peptic ulcer bleeding are non-steroidal anti-inflammatory drugs (NSAIDs) use and *Helicobacter pylori* infection.

More recently, selective serotonin reuptake inhibitors (SSRIs) have been identified as another risk factor [2], and since then, 15 additional studies –including this one– addressing this topic have been carried out [3]–[16]. Albeit four studies found a strong significant association between SSRIs and upper GI bleeding (a risk value higher than 2) [2], [5], [7], [11], others found no association at all [3], [8], [10]; thereby, the association remains a matter of controversy.

The widespread use of antidepressants, particularly SSRIs, makes even small risks account for a large number of cases, converting this problem into an important public health issue. This fact, along with the lack of consistency of the findings in the studies carried out so far, has aroused a great interest on this subject. Our study has been conducted with carefully collected information to further understand the relationship between SSRIs and upper GI bleeding in the framework of a general study on risk factors of upper GI bleeding.

## Methods

### Study design

We conducted a, multicentre, case-control study in 4 hospitals in Spain and 1 in Italy. Patients were recruited from January 2004 to July 2006 in Spain and from October 2005 to November 2007 in Italy; the population covered by these hospitals was 1,570,687 inhabitants.

### Cases and controls

Records of all endoscopic procedures and lists of admission diagnosis in the participating hospitals were examined daily. Cases were patients aged ≥18 years who were admitted with a primary diagnosis of acute upper GI bleeding from a duodenal or gastric ulcer, acute lesions of the gastric mucosa, erosive duodenitis or mixed lesions, all of them diagnosed by endoscopy; patients with endoscopic diagnosis other than bleeding from the above specified lesions were excluded from the study. For each case, up to 3 controls ≥18 year-old, matched by sex, age (±5 years), date of admission (within 3 months) and hospital were selected; they were recruited from patients who were admitted for elective surgery for non-painful disorders, including inguinal hernia, prostate adenoma and cataracts. According to the null hypothesis approach, they were expected to have a prevalence of drug use similar to that of the underlying population from which the cases arise. All subjects, regardless of their condition of case or control, who at the start date had a history of cancer, coagulopathy, Mallory-Weiss syndrome and esophageal varices, were excluded; those who were non-residents in the study area and those with no reliable interview were also excluded. An upper GI bleeding odds ratio of 2 could be detected with a power of 80% with 500 cases and 1500 controls (crude power calculation), assuming a prevalence of SSRI use of 4% (alpha error 5%).

### Information retrieval and exposure definition

After obtaining an informed consent, especially trained monitors interviewed patients with a structured questionnaire within 15 days of admission; this questionnaire had several questions about previous use and frequency of use of medicines, with a series of colour pictures including the agents of interest. The interview included detailed information on symptoms leading to the current admission, and on the clinical and medication history.

Exposures to SSRIs, other antidepressants and other drugs were defined as any use of these drugs which lasted until the index date or when discontinued within 7 days before the index date (the day on which first symptoms of upper GI bleeding started). For each case, the index date was defined blindly to the use of drugs; consumption of medications between index dates and interview dates was not taken into account for the purpose of defining exposures (i.e., comsumption of medications could be altered by the presence of the first signs of disease). For controls, index date was the day of the interview.

To estimate the association with upper GI bleeding, different groups were established. As a first approach, antidepressants were considered all together; then, antidepressants were classified into three groups according to their affinity for the serotonin transporter, i.e., high affinity (fluoxetine, paroxetine, sertraline and clomipramine), intermediate affinity (amitriptyline, fluvoxamine, citalopram, imipramine, dosulepin, venlafaxine, duloxetine, escitalopram and melitracen) and low affinity (mirtazapine, nortriptyline, desipramine, trimipramine, maprotiline, trazodone, mianserin, amoxapine, bupropion, doxepin, moclobemide, ciclobenzaprine and etoperidone) [17]; lastly, antidepressants were classified according to selectivity, which in turn was based on the ratio of the equilibrium dissociation constants for the serotonin over the noradrenaline transporter; thus, for SSRIs (ATC class N06AB), sertraline, fluoxetine, fluvoxamine, paroxetine, citalopram, etoperidone and escitalopram were grouped; for non-SSRIs, amitriptiline, dosulepin, trimipramine, doxepine, maprotiline, amoxapine, imipramine, nortriptiline and clomipramine were grouped; and for other antidepressants, the rest of active ingredients were grouped.

### Main outcome measures and statistical analysis

Adjusted odds ratios (OR) and their 95% confidence intervals (95% CI) were estimated by means of a generalised linear mixed model for dependent binomial-type variables (case or control) [18]. This approach has three advantages over the application of conditional logistic regression: (i) it takes into account the multicentre character of the study when the models are developed; (ii) it allows to take advantage of the information of the strata (each case and its matched controls) containing only cases or controls; and (iii) all members have identical values for the covariates [18].

In the construction of the model, patients were taken as level one, strata as level two, and hospitals as level three. In the estimation of the models we used the lmer function, implemented in the context of the lme4 R package (version 2.7.2) [19]. This function performs the fit by using the Laplace approximation, and the correlation structure within the random effects has only an additional constraint: the variance-covariance matrix must be symmetric and positive semidefinite [20].

To construct these models, the absence or lowest level of exposure was taken as the reference category. We performed a bivariate analysis, using the variables of exposure and potential confounders; and a multivariate analysis, including those independent variables which yielded a statistical significance of less than 0.2 in the bivariate analysis. The independent variables with the highest level of statistical significance were successively eliminated from the original model, provided that the coefficients of the principal variables of exposure changed by no more than 10% and Schwarz's Bayesian Information Criterion.

In addition to each antidepressant group, the following variables were included in the model: alcohol and caffeine consumption, past history of GI disorders, family history of GI bleeding, osteoarthritis, number of medicines taken and the use of NSAIDs, salicylates (analgesic doses), proton pump inhibitors, H2 antihistamines, antacids, antiplatelet agents and anticoagulants (only vitamin K antagonists).

The protocol of the study was approved by the corresponding Ethics Committees of the participating hospitals. All patients were asked for an informed consent and, to be included in the study, they had to give a written informed consent.

## Results

Out of 4325 patients who were interviewed, 1939 were finally considered eligible for the study: 581 cases of upper GI bleeding and 1358 controls; 2386 patients were excluded for not meeting the case or control definition (2169 cases and 217 controls) and 146 (32 cases and 114 controls) explicitly refused to participate. Frequent clinical signs of the cases were: melena (86.3%), dizziness (59.2%), asthenia (56.3%) or hematemesis (40.7%). Corresponding controls were 704 (51.8%) patients admitted for cataract surgery and 329 (24.2%) patients with non painful inguinal hernias; the remaining were patients admitted for varicectomy, prostate adenoma surgery, septoplasty, lipoma removal and others. The median time from hospital admission until interview was 2 days for cases and 0 days for controls.

Distribution of prognostic factors between cases and controls after matching is presented in Table 1. No differences in age or sex distribution were observed between cases and controls after matching. Overall, 4.0% of the cases and 3.3% of controls used an SSRI antidepressant in the week before the index day. No significant risk of upper GI bleeding was encountered for SSRI antidepressants (adjusted OR, 1.06, 95% CI, 0.57–1.96) or for whichever other grouping of antidepressants (Table 2).

**Table 1:** Baseline characteristics.

	Number (%)		
	Cases(n = 581)	Controls(n = 1358)	Crude OR(95% CI)
Age (mean; SD)	62.6; 17.0	63.2; 15.7	Matching factor
Females	152 (26.2)	405 (29.8)	Matching factor
BMI (mean; SD)^a^	26.6; 4.3	26.6; 4.0	
Study years^b^	8.4; 4.5	7.9; 4.2	
Smoking status			
*Non-smoker*	249 (42.9)	635 (46.8)	1 (reference)
*Ex-smoker*	193 (33.2)	445 (32.7)	1.11 (0.88–1.38)
*Current smoker*	139 (23.9)	278 (20.5)	1.28 (0.99–1.64)
Caffeine consumption	490 (84.3)	1148 (84.5)	0.98 (0.75–1.29)
Alcohol intake^c^			
*No intake*	197 (33.9)	475 (35.0)	1 (reference)
*Mild*	255 (43.9)	658 (48.5)	0.93 (0.75–1.16)
*Moderate*	99 (17.0)	196 (14.4)	1.22 (0.91–1.63)
*Heavy*	30 (5.2)	29 (2.1)	2.49 (1.46–4.27)
Family history of GI bleeding	150 (26.6)	229 (17.5)	1.71 (1.35–2.16)
Previous history of GI tract disorders			
*None*	225 (38.9)	660 (48.9)	1 (reference)
*Dyspepsia*	149 (25.8)	498 (36.9)	0.88 (0.69–1.11)
*Ulcer*	81 (14.0)	106 (7.8)	2.24 (1.62–3.11)
*Bleeding*	123 (21.3)	87 (6.4)	4.15 (3.03–5.67)
Co-morbidity			
*Diabetes*	95 (16.4)	187 (13.8)	1.22 (0.93–1.60)
*Heart disease*	153 (26.8)	280 (20.8)	1.39 (1.11–1.75)
*Hypertension*	227 (39.1)	509 (37.8)	1.06 (0.87–1.29)
*High cholesterol*	174 (30.3)	381 (28.4)	1.10 (0.89–1.36)
*Osteoarthritis*	169 (30.7)	445 (35.0)	0.83 (0.67–1.02)
Intake of more than 3 medications	310 (53.4)	565 (41.6)	1.61 (1.32–1.95)

Distribution of cases and controls according to prognostic factors.

Abbreviations: OR, odds ratio; CI, confidence interval.

a For Body Mass Index (BMI) there were 579 cases and 1354 controls available.

b For study years there were 578 cases and 1354 controls available.

c Alcohol consumption: In women: low, ≤20 mg/day; moderate, between >20 mg/day and ≤60 mg/day; high, >60 mg/day. In men: low, ≤30 mg/day; moderate, between >30 mg/day and ≤80 mg/day; high, >80 mg/day.

**Table 2:** Risk of upper gastrointestinal bleeding associated with the intake of antidepressants.

	Number (%)			
Category^a^	Cases(n = 581)	Controls(n = 1358)	Crude OR (95% CI)^b^	Adjusted OR (95% CI)^c^
All antidepressants	33 (5.7)	74 (5.4)	1.04 (0.68–1.59)	0.91 (0.54–1.52)
High affinity	15 (2.6)	34 (2.5)	1.03 (0.56–1.91)	0.91 (0.43–1.93)
Intermediate affinity	17 (2.9)	36 (2.7)	1.11 (0.62–1.99)	1.11 (0.54–2.28)
Low affinity	3 (0.5)	12 (0.9)	0.58 (0.16–2.07)	0.48 (0.12–1.94)
SSRIs	23 (4.0)	45 (3.3)	1.20 (0.72–2.01)	1.06 (0.57–1.96)^d^
Non-SSRIs	7 (1.2)	23 (1.7)	0.71 (0.30–1.66)	0.87 (0.32–2.39)
Other antidepressants	6 (1.0)	15 (1.1)	0.93 (0.36–2.42)	0.45 (0.14–1.46)

Abbreviations: OR, odds ratio; CI, confidence interval.

a Antidepressants:

High affinity: fluoxetine, paroxetine, sertraline and clomipramine.

Intermediate affinity: amitriptyline, fluvoxamine, citalopram, imipramine, dosulepin, venlafaxine, duloxetine, escitalopram and melitracen.

Low affinity: mirtazapine, nortriptyline, desipramine, trimipramine, maprotiline, trazodone, mianserin, amoxapine, bupropion, doxepin, moclobemide, ciclobenzaprine and etoperidone.

SSRIs: sertraline, fluoxetine, fluvoxamine, paroxetine, citalopram, etoperidone and escitalopram.

Non-SSRIs: amitriptiline, dosulepin, trimipramine, doxepine, maprotiline, amoxapine, imipramine, nortriptiline and clomipramine.

Other antidepressants: bupropion, ciclobenzaprine, desipramine, duloxetine, melitracen, mianserin, mirtazepine, moclobemide, trazodone and venlafaxine.

b Adjusted for matching factors: age, sex (±5 years), date of admission (within 3 months) and hospital.

c Adjusted for alcohol and caffeine consumption, past history of GI disorders, family history of GI bleeding, osteoarthritis, number of medicines taken and use of NSAIDs, salicylates (analgesic doses), proton pump inhibitors, H2 antihistamines, antacids, antiplatelet agents and anticoagulants (only vitamin K antagonists).

d When applying conventional logistic regression, the adjusted estimate for SSRIs was 1.24 (95%CI, 0.62–2.48).

The proportion of patients exposed to NSAIDs was 21.9% for cases and 7.6% for controls; adjusted OR for NSAIDs was 4.36 (95% CI, 3.11–6.13). Prevalence of self-medication with NSAIDs or analgesic doses of salycilates was 7.5% (cases, 15.5%; controls, 4.1%); this was slightly more frequent in those having SSRI antidepressants than in those not having these medications (8.8% vs. 7.5%). Self-medication with NSAIDs or analgesic doses of salycilates had no influence on final estimates; when the analysis was restricted to non self-medicated patients, the risk of upper GI bleeding associated with SSRIs neither was modified (adjusted OR, 0.97, 95% CI, 0.50–1.87). Eight patients were simultaneously taking SSRIs and NSAIDs (4 cases and 4 controls).

Acid-suppressing drugs (proton pump inhibitors or H2 antihistamines) were taken by 11.9% of the cases and 17.1% of the controls; the adjusted ORs for proton pump inhibitors were 0.42 (95% CI, 0.29–0.63) and 0.33 (95% CI, 0.17–0.65) for H2 antihistamines. Cases and controls receiving SSRIs had also more acid-suppressing drugs than non-SSRI users (22.1% vs. 15.3%). Similarly, when the analysis was restricted to those patients not taking acid-suppressing drugs, results did not change (SSRIs adjusted OR for upper GI bleeding, 1.11, 95% CI, 0.56–2.18).

## Discussion

When our study protocol was first prepared, four studies had been published [2]–[5]; three of those studies found odds ratios of 3 or more and only one, clearly heterogeneous in design, found no risk [3]; thus, our study had power to detect an odds ratio of 2 and failed to detect an association between SSRI exposure and occurrence of upper GI bleeding of this magnitude; in it, we have found a clear association with NSAIDs in the range of that reported in the literature [21]. When considering the different groupings of antidepressants there were no differences. This is consistent with the results of another case-control study similarly conducted with the same protocol and in a close similar healthcare setting [10]. Out of 15 studies conducted so far (Table 3), 4 found a strong significant association (a relative risk value higher than 2) [2], [5], [7], [11], 5 found a significant mild association (a risk value between 1 and 1.7) [9], [12], [13], [15], [16] and 5 did not find any association at all [3], [8], [10], [14, present study]; in the remaining two studies, the outcome variables were not entirely comparable to those of the other studies, but a trend to an increased risk was observed with increased serotonin inhibition reuptake [4], [6]. Since all these studies have been conducted during different periods, in different geographical areas and healthcare settings, with different designs and different ways of collecting information, the explanation of those different estimates may lie on this heterogeneity; nevertheless, retrospective or prospective character seems to be a remarkable factor of heterogeneity (Table 3); thus, only 1 out of 4 prospective studies published so far found a significant risk while, in the retrospective studies, it was 9 in 12 which found a significant risk. It is possible that, since most of these retrospective studies have been conducted with pre-existing information –not collected for the purposes of these particular studies– some relevant confounding factors might be difficult to adjust for. For instance, in these studies neither there is information upon intake of medication, nor upon exposure to OTC drugs –depressive patients are more prone to self-medicate [22], nor upon co-morbidities not stated that may lead to the intake of non-prescription NSAIDs, nor, in some studies, upon alcohol intake. In our study, we could control for self-medication with NSAIDs; thus, when restricted to non self-medicated patients, the risk slightly decreased denoting a certain influence of self-medication. However, it cannot be ruled out that self-medication, with a differential distribution between cases and controls, might have some influence on other studies. Furthermore, it has been pointed out that observational studies, in which cases are collected from hospitals and controls are non-hospitalised patients, might be affected by a selection bias [23]. In the study by de Abajo et al. [12], the prevalence of current use for acid-suppressing drugs (proton pump inhibitors or H2 antihistamines) was 19.5% in case patients and 11.2% in controls (11.9% and 17.1%, respectively, in our study); therefore, the authors state that these figures suggest an important confounding factor by indication; in a broader approach, they might be also interpreted as a risk marker denoting differential severity between cases and controls impossible to fully adjust for in that study (crude estimate for acid-suppressing drugs was 2.0 and 1.2 after adjustment). Selection bias is consistent with the fact that none of the studies in which cases and controls came from hospitals found a significant risk [8], [10], [14, present study]. Thus, a possible explanation for the risk detected in some studies would be that, since depression is currently associated with more morbidity and also with past use of antidepressants, these drugs in turn might be spuriously associated with bleeding.

**Table 3:** Epidemiological studies assessing the association between SSRIs exposure and the occurrence of upper GI bleeding.

Study/year	n	Adjusted OR (95% CI)
*Retrospective* a		
de Abajo et al.,1999 [2]	11,651	3.0 (2.1–4.4)
Tata et al., 2005 [7]	64,417	2.4 (2.1–2.7)
Van Walraven et al., 2001 [4] b c d	317,824	3.0 (2.6–3.6)
Dalton et al., 2003 [5] c	26,005	3.6 (2.7–4.7)
Wessinger et al., 2006 [8] b d	1,579	1.3 (0.8–1.9)
Meijer et al., 2004 [6] d e f	1,003	2.1 (0.6–8.3)
Helin-Salmivaara et al., 2007 [9]	50,971	1.3 (1.1–1.5)
De Abajo et al., 2008 [12]	11,321	1.6 (1.2–2.1)
Opatrny et al., 2008 [13]	44,199	1.3 (1.1–1.6)
Barbui et al., 2009 [14] c	2,998	1.3 (0.9–1.9)
Targownik et al., 2009 [15]	70,142	1.4 (1.1–1.9)
Dall et all., 2009 [16]	36,852	1.7 (1.5–1.9)
*Prospective* a		
Dunn et al., 2000 [3] c	237,609^g^	1.2 (0.9–1.7)
Vidal et al., 2008 [10] e	9,841	1.2 (0.9–1.7)
Lewis et al., 2008 [11] c	2,245	2.1 (1.3–3.3)
Present study, 2009^e^	1,939	1.1 (0.6–2.0)

a For the purpose, a retrospective study is when the idea for the study was developed after data collection; the opposite is termed prospective.

b These studies were based in America, the rest in Europe.

c Cohort studies.

d The outcome variables were not entirely comparable to those of the other studies.

e In these studies, cases and controls were recruited from hospitals.

f Estimated risk is for high-affinity serotonin inhibitor antidepressants versus low-activity ones.

g Patient-months.

Had the SSRIs caused upper GI bleeding through a mechanism related to serotonin reuptake inhibition, other bleeding complications might also appear; however, there is no consistency at this regard. While two epidemiological studies upon abnormal bleeding and perioperative blood transfusion, respectively, suggested an increased risk related to SSRIs [6], [24], in other 6 studies upon hemorrhagic stroke [25]–[28], postpartum haemorrhage [29] and hemorrhagic events in different locations [30], no association was found.

Depletion of serotonin from platelets caused by SSRIs has been currently postulated as the most likely mechanism for bleeding [31]. Accordingly, inhibition by SSRIs of serotonin reuptake by platelets is thought to lead to reduced platelet serotonin levels and it would lead to diminished serotonin release from platelets on activation and to decreased platelet aggregation. In the study by Hergovich et al. [32], serotonin concentration in the platelets decreased by 83% after 14 days of paroxetine use (20 mg/day) and the platelet function analyser (PFA)-closure time was increased by 31%. Conversely, the results of other studies that have also investigated the effect of SSRI treatment on platelet activity are not consistent. McCloskey et al. [33], in a study comparing patients treated with SSRIs (n = 32) and patients on bupropion (n = 29), found significant platelet abnormalities by using platelet aggregation and release assays but not when using the PFA-100 method. In another study, only 6 out of 43 patients treated with different SSRIs had an abnormal platelet function (PFA-closure time >150 s) [34]. A third study, comprising 12 healthy young men, did not find any difference between sertraline and placebo intake regarding platelet activity and serotonin uptake [35]. Furthermore, there is no a clear correlation between clinical bleeding and platelet aggregation abnormalities; in a series of 35 patients with high-abnormal bleeding time, 21 (60%) had a normal platelet function [36]. Recently, it has been suggested that SSRIs added to platelet impaired function may have a direct harmful effect on the GI tract mucosa based on observations by Takeuchi et al. [37]. These authors did observe that paroxetine worsens the development of antral ulcers induced by indomethacin in rats; however, in the same experiments, it was similarly observed that paroxetine, dose-dependently suppressed indomethacin-induced gastric corpus and intestinal lesions, which precludes any firm conclusions and requires further research.

The stringent definition of exposure, the carefully and thorough information gathered and the objective ascertainment of the cases are the main strength of our study; also, by using prompt cards –a series of colour pictures including the drugs of interest, we could avoid or highly reduce recall bias. Additionally, the statistical analysis performed showed that the estimates values found by applying a generalised linear mixed model or the conventional logistic regression were grossly coincidental (footnote on Table 2). On the contrary, the small percentage of patients having SSRIs and NSAIDs simultaneously prevents the analysis of interaction; also, the sample size does not permit to analyse by certain subgroups or individual drugs. It is possible as well that, by using hospitalized controls, we selected a sicker group as comparator; however, the prevalence of SSRIs use among controls in our study is close similar to figures for the general population in Spain [38]. Moreover, considering prevalence of acid-suppressing drugs as a risk marker and assuming an imbalance in severity between our cases and controls, it could be fully dealt with in the analysis since we found expected estimates for acid-suppressing drugs when adjusting (adjusted OR for proton pump inhibitors, 0.42, 95% CI, 0.29–0.63; adjusted OR for H2 antihistamines, 0.33, 95% CI, 0.17–0.65); likewise, the risk estimate for NSAID-induced upper GI bleeding was as expected. Another limitation would be the possibility that patients in the study population with emergency bleeds would go to different hospitals, and then undergo elective surgeries; however, the base population is the same for both as the elective surgery hospitals belong to the same population area covered by the main hospitals. In addition, patients cannot select the hospital where the surgical intervention will be performed; this is planned by the National Health Service.

In summary, the results of this case-control study showed no significant increase in upper GI bleeding with SSRIs and provide good evidence that the magnitude of any increase in risk is not greater than 2.
